# High rate of progression to symptomatic multiple myeloma in patients with smoldering myeloma and isolated osteoporotic vertebral fracture

**DOI:** 10.1016/j.bonr.2024.101755

**Published:** 2024-03-25

**Authors:** Kevin Chevalier, Sabrina Hamroun, Samuel Bitoun, Julien Henry, Christian Roux, Karine Briot, Rakiba Belkhir, Xavier Mariette, Raphaèle Seror

**Affiliations:** a Department of Rheumatology, Université Paris-Saclay, Assistance Publique-Hôpitaux de Paris (AP-HP), Hôpital Bicêtre, Le Kremlin-Bicêtre, France; b Department of Rheumatology, Université Paris-Cité, Assistance Publique-Hôpitaux de Paris (AP-HP), CHU Cochin, Paris, France

**Keywords:** Osteoporosis, Smoldering myeloma, Vertebral fracture

## Abstract

Multiple myeloma (MM) frequently causes vertebral fractures (VF). Some are lytic lesions and others have the aspect of benign osteoporotic fractures not requiring anti-myeloma treatment. We explored outcome of these patients with smoldering myeloma (SM) and osteoporotic VF.

In this retrospective bi-centric study, patients were identified using a systematic keyword search on electronic medical records. Patients with SM and isolated VF of osteoporotic aspect without indications for myeloma-specific therapy were included.

Overall, 13 (7 %) of the 184 identified patients had SM and VF confirmed to be osteoporotic (median number of VF was 3). During follow-up, 12 (92 %) patients evolved to symptomatic MM, 7 (54 %) of them within 18 months (early progressors). Myeloma defining events were new lytic bone lesions in 7 patients (53.8 %). The serum calcium level was significantly higher in the early progressor group (median 2.35 IQR [2.31–2.38] and 2.28 IQR [2.21–2.29] respectively, *p* = 0.003). Early progressors had a higher number of VF at diagnosis (3.0 [2.0–5.5] vs 1.0 [1.0–2.5], *p* = 0.18) and more frequently evolved to symptomatic MM because of lytic bone lesions (5 [71 %] vs 2 [33 %], *p* = 0.13) compared to late progressors.

VF of osteoporotic appearance in the context of SM is a rare situation but at high risk of rapid progression to symptomatic MM, suggesting that they may represent bone fragility linked to MM infiltration rather than solely osteoporotic fractures. Further studies are needed to assess if earlier treatment might be beneficial in this population.

## Highlights

• Progression is frequent in patients with vertebral fractures and smoldering myeloma
• Half of them progress within 18 months after vertebral fracture diagnosis
• Prospective studies are needed to determine the prognosis of these patients

## Graphical abstract

**Figure:**
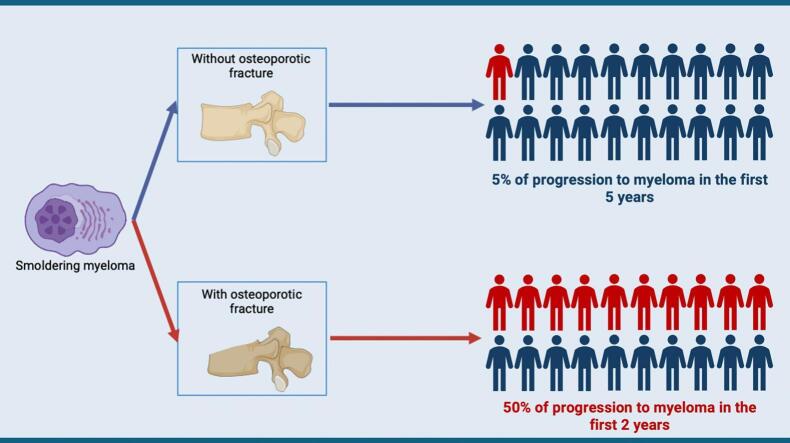

## 1 Introduction

Multiple myeloma (MM) is a cytogenetically heterogeneous clonal plasma cell proliferative disorder defined by clonal bone marrow plasma cells ≥10 % or a biopsy-proven osseous or extramedullary plasmacytoma and any one or more of the following myeloma-defining events: hypercalcemia, renal failure, anemia or bone lesions (referred to as CRAB features); a clonal bone marrow plasma cell percentage ≥ 60 %; an involved/uninvolved serum free light chain ratio > 100 or > 1 focal lesion on magnetic resonance imaging (MRI) studies (Rajkumar et al., 2014). MM is almost always preceded by an asymptomatic premalignant phase called monoclonal gammopathy of undetermined significance (MGUS) (Rajkumar et al., 2014). MGUS is a common condition in the population over 50 years of age, affecting 3–4 % of this population and requiring no treatment. Therefore, one of the main questions is how to differentiate MGUS from MM. The diagnosis of MGUS requires the absence of MM criteria. Between these two states, there exists an intermediate stage called smoldering myeloma (SM) defined by a clonal bone marrow plasma cells between 10 and 60 %, or a serum monoclonal protein (IgG or IgA) ≥30 g/L or an urinary monoclonal protein ≥500 mg per 24 h without any myeloma-defining events and did not require initiation of treatment (Rajkumar et al., 2014).

Thus, lytic bone lesions are a myeloma-defining criterion in the 2014 revision of the IMWG (International Myeloma Working Group) criteria (Rajkumar et al., 2014). However, osteoporotic vertebral fractures (VF) are not a lytic lesion and should not be considered a myeloma-defining event. Approximately one third of fractures in MM occur as fragility fractures at tumor-free sites (Melton et al., 2004). As a consequence, Nador et al. recommended not to treat MM without CRAB criteria in cases of fragility fracture (Nador et al., 2019).

When comparing acute osteoporotic VF in MGUS and MM patients, no difference was found in the number of VF, bone mineral density, or bone turnover suggesting that osteoporotic VF is not a MM-defining event (Golombick and Diamond, 2008). However, there are no data on the prognosis and outcome of patients with SM who have VF that are thought to be of osteoporotic origin. Indeed, the pure osteoporotic nature of VF is sometimes difficult to establish. In addition, the risk of progression to symptomatic MM in these patients is largely unknown. Therefore, the aim of this study was to evaluate the outcome of patients with SM and osteoporotic-like VF and to identify factors associated with rapid progression to symptomatic MM.

## 2 Material and methods

### 2.1 Patient's selection

In this retrospective, observational, bicentric study, patients were identified through a systematic search of the electronic medical record database of all patient's files of the tertiary care rheumatology departments of the Bicêtre and Cochin hospitals (Assistance Publique – Hopitaux de Paris (AP-HP), France), specialized in the care of MM patients. Since February 2013, medical records in all departments of the hospitals are fully electronic. We identified patients with MM using the International Classification of Diseases 10th Revision (ICD-10) codes: “multiple myeloma and plasma cells malignant tumor” (C90) whose chart contained any of the following keywords: “osteoporosis”, “osteoporotic” or “porotic” between February 2013 and May 2021. Patients were included if they had at least, one radiologically confirmed osteoporotic VF. A radiologist experienced in osteoarticular imaging at both centers determined the benign nature of the VF. Osteoporotic fractures were defined as: Fractures with puzzle sign, Sharp fractures lines, intervertebral vacuum phenomenon, fractures without lytic lesion, without epidural soft tissue mass as defined by Mauch et al. (2018). All patients fulfilled the IMWG 2014 criteria for SM at the time of the acute VF. Patients treated for MM at the time of VF diagnosis were also excluded. Data collection and ethical approval are detailed in the supplementary material.

Data collection and ethical approval are described in detail in the Supplementary Appendix.

### 2.2 Statistical analysis

Data are expressed as median [interquartile range; IQR] and number (%). Patient characteristics were compared between those requiring a treatment before and after 18 months from the diagnosis of VF. Comparisons were made using Wilcoxon test for quantitative parameters and Fischer's exact test for qualitative data. A *p*-value <0.05 was considered as statistically significant. All analyses were performed with R Studio V.4.0.3.

## 3 Results

### 3.1 Patient selection and description

**Fig. 1:**
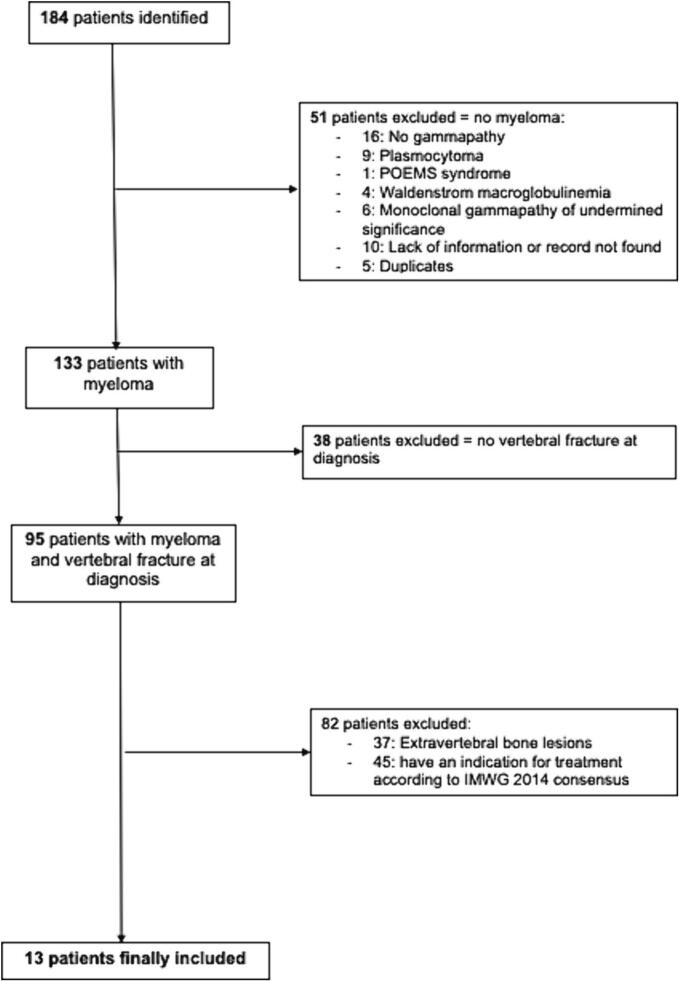
Flow chart.

**Fig. 2:**
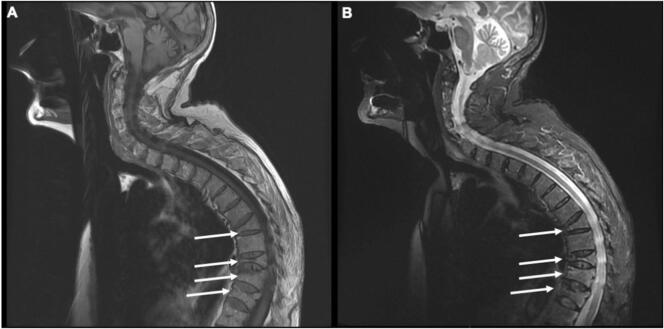
Multiple vertebral fractures(arrows) with osteoporotic appearance without myeloma defining criteria in T1-weighted (A) and T2 STIR-weighted sequences (B) in a patient with smoldering myeloma.

The electronic search identified 184 patients, of whom 95 had MM and at least one VF of osteoporotic appearance at diagnosis (Fig. 1). After exclusion of patients who had an indication for initiation of MM treatment (i.e., a myeloma defining events according to the revised IMWG diagnostic criteria (1)) at diagnosis, 13/184 (7 %) patients with SM and VF were finally included (Table 1). They had a median of 3 [1.0–5.0] VFs. An alternative cause of secondary osteoporosis was found in 5 patients (39 %) (supplementary table s1). Bone mineral density was available for 7 patients, of whom 3 had a spinal osteoporosis, and none had a femoral osteoporosis. All patients had a whole spine MRI. It showed a characteristic aspect of osteoporotic VF, no lytic or modular lesion and no other spinal lesion (Fig. 2). An aspect of spinal infiltration was observed in two patients. Looking at the Mayo Clinic's 20/20/20 Risk Model, 9, 4 and 0 patients in our study were at low, intermediate, and high risk, respectively. Of the patients at intermediate risk, 3 were at intermediate risk due to a serum M-protein level > 20 g/L and 1 due to a serum free light chain ratio > 20. Of the early progressors, only 1 was intermediate risk and all the others were low risk.

**Table 1:** Patients' characteristics.

	Patients (n = 13)
Age (years), median [IQR]	72.0 [66.0–77.0]
Gender n (%) Female	6 (46)
At SM diagnosis	
ISS score at diagnosis	
ISS 1, n (%)	11 (85)
ISS 2, n (%)	1 (8)
ISS 3, n (%)	0 (0)
Serum M protein level (g/L), median [IQR]	15.4 [13.6–22.2]
IgG isotype n (%)	12 (92)
IgA isotype n (%)	1 (8)
Light chain n (%) Kappa	8 (62)
Free light chain ratio, median [IQR]	30 [21.1–69.5]
Total gammaglobulin (g/L), median [IQR]	20.4 [19.3–28.5]
Residual gammaglobulin (g/L), median [IQR]	4.8 [3.8–5.3]
Percentage of Plasma cells on bone aspiration, median [IQR]	12.0 [10.0–17.0]
Plasma cells on bone aspiration n (%)	13 (100)
Calcemia (mmol/L), median [IQR]	2.31 [2.29–2.35]
Creatinine (μmol/L), median [IQR]	74.0 [58.0–84.8]
Hemoglobinemia (g/dL), median [IQR]	11.7 [10.5–12.6]
Proteinuria (g/24 h), median [IQR]	0.06 [0.00–0.13]
Number of vertebral fractures, median [IQR]	3.0 [1.0–5.0]
Localisation of vertebral fractures	
Thoracic n (%)	4 (31)
Lumbar n (%)	6 (46)
Thoracic and lumbar n (%)	3 (23)
25-OH vitamin D (ng/mL), median [IQR]	35.0 [15.0–42.0]
Secondary osteoporosis n (%)	5 (39)
Time between smoldering myeloma and myeloma treatment or loss of follow-up (months), median [IQR]	17.0 [13.0–29.0]
Total follow up duration (months), median [IQR]	71.0 [46.8–101.5]
Time without myeloma treatment < 12 months n (%)	3 (23)
Time without myeloma treatment < 18 months n (%)	7 (54)
Osteoporosis treatment n (%)	10 (76.9)
Immunoparesis (g/L), median [IQR]	3.1 [2.6–4.3]

IQR: Interquartile range; ISS: International Staging System; MRI: magnetic resonance imaging; SM: Smoldering myeloma.

In total, 11/13 (85 %) patients received bisphosphonates: intravenous in 10 (annual in 8, bi-annual in 1, and monthly in 1) and oral in 1. Not all patients with initiation of osteoporotic treatment had densitometric osteoporosis so the vertebral fracture was the indication of treatment initiation.

After a median follow-up of 71 months [IQR:46.0–101.5], 12/13 (92 %) patients eventually progressed to symptomatic MM and received treatment. The median time to progression and treatment initiation was 17.0 [IQR:13.0–29.0] months, with a maximum of 137 months. Seven (54 %) patients progressed and were treated within the 18 months of the diagnosis of the osteoporotic VF (early progressors) and 6 (46 %) were treated ≥18 months later (late progressors). Of note, 5 patients had a second bone marrow aspiration, median 25 [16–29] months after the first. In all but one patient, the rate of plasma cell infiltration increased between the two biopsies (median plasma cell infiltration increased by 18 [6–19] %).

Treatment initiation for MM was mainly driven by the occurrence of lytic bone lesions (7 patients, 54 %), anemia (5 patients, 39 %), and increased serum free light chain ratio > 100 (3 patients, 23 %). Four patients (31 %) had more than one treatment-related criterion. One patient had hypercalcemia.

### 3.2 Identification of factors of early progression

We did not find any predictive factor of early progression except a significantly higher serum calcium level (although still in the normal range) in early progressors (median: 2.35 IQR [2.31–2.38] versus 2.28 IQR [2.21–2.29] (*p* = 0.003) (Table 2)).

**Table 2:** Comparison of baseline characteristics of patients with smoldering myeloma and frailty vertebral fracture between early (<18 months) and late progressors (>18 months) to symptomatic multiple myeloma.

	Late progressors > 18 months n = 6	Early progressors < 18 months n = 7	p-value
Female gender n (%)	3 (50.0)	3 (43)	1
Age (years), median [IQR]	69.0 [66.8–71.3]	77.0 [68.0–79.0]	0.389
ISS at diagnosis, n (%)			1
ISS 1	5 (83)	6 (86)	
ISS 2	1 (17)	0 (0)	
Serum M protein level at diagnosis (g/L), median [IQR]	20.6 [15.0–26.6]	15.0 [12.4–16.7]	0.445
Total gammaglobulin at diagnosis (g/L), median [IQR]	24.1 [20.4–32.8]	19.6 [18.7–22.4]	0.247
Residual polyclonal gammaglobulin at diagnosis (g/L), median [IQR]	4.8 [1.9–6.8]	4.9 [4.3–5.4]	0.931
IgG Isotype n (%)	6 (100)	6 (86)	1
Free Light chain Kappa n (%)	4 (67)	4 (57)	1
Plasmocytes on aspiration at diagnosis, (%) median [IQR]	16.5 [11.5–17.8]	11.0 [9.0–14.0]	0.389
Calcemia at diagnosis (mmol/L), median [IQR]	2.28 [2.21–2.29]	2.35 [2.31–2.38]	0.034
Creatinine at diagnosis (μmol/L), median [IQR]	80.0 [46.0–80.0]	69.0 [63.5–89.0]	1
Hemoglobinemia at diagnosis (g/dL), median [IQR]	12.0 [11.0–12.9]	11.5 [10.5–12.2]	0.626
Proteinuria at diagnosis (g/24 h), median [IQR]	0.09 [0.00–0.14]	0.05 [0.00–0.06]	0.829
Number of vertebral fractures, median [IQR]	1.0 [1.0–2.5]	3.0 [2.0–5.5]	0.176
Vertebral infiltration in MRI, n (%)	0 (0)	2 (29)	0.462
25 OH vitamine D (ng/mL), median [IQR]	35.0 [19.0–42.0]	32.5 [14.8–41.3]	1
Secondary osteoporosis n (%)	2 (33)	3 (43)	1
Osteoporosis treatment (PO/IV) n (%)	5 (83)	5 (71)	1
Myeloma defining event			
Elevation of free light chain ratio > 100 n (%)	2 (33)	1 (14)	0.553
New lytic bone lesion n (%)	2 (33)	5 (71)	0.126
Anemia n (%)	3 (50)	2 (29)	0.558

PO: Per os; IQR: Interquartile range; IV: intravenous; ISS: International Staging System, MRI: magnetic resonance imaging.

Nevertheless, early progressors had a higher number of VFs at the time of SM diagnosis (median: 3.0 [2.0–5.5] vs. 1.0 [1.0–2.5], *p* = 0.176) and more frequently progressed to symptomatic MM due to lytic bone lesions (5 [71 %] vs. 2 [33 %], *p* = 0.13) compared to late progressors. Also, both patients with diffuse vertebral infiltration on MRI were early progressors. However, these differences were not significant due to the small sample size.

## 4 Discussion

MM and osteoporosis mainly affect patients after 60 years of age, and both diseases may occur independently. Our study suggests that in when a patient presents with vertebral fractures with osteoporotic aspect and SM this should raise the clinician's concern. Even when VF was of osteoporotic origin, all but one patient developed symptomatic MM requiring treatment initiation after a median of 17 months.

The association between MGUS and bone fragility has been well documented in the literature, with an increased risk of VF and/or osteoporosis in MGUS patients compared to controls (Golombick and Diamond, 2008; Gregersen et al., 2006; Bida et al., 2009; Kristinsson et al., 2010; Sfeir et al., 2020; Ng et al., 2011). Similarly, the association between MM and low bone mineral density has been less studied (Mariette et al., 1992).

The originality of our study is to describe the outcome of patients with SM and isolated osteoporotic VF without other criteria for MM treatment initiation. This situation represents a difficult clinical situation because the osteoporotic character of the VF may be difficult to certify. In our study, the osteoporotic nature of the lesion was determined by trained radiologists and by MRI performed in all patients.

The rate of progression (50 % at 17 months) was much higher than expected in SM (10 % per year during the first 5 years) (Kyle et al., 2007; Kyle et al., 2014). Interestingly, the most common myeloma-defining event was the appearance of a lytic bone lesion, especially in early progressors. We also observed that early progressors had a numerically higher number of VFs at the time of SM diagnosis. Taken all together, these data suggest that osteoporotic VF, especially when multiple, may represent bone fragility associated with MM infiltration rather than osteoporotic lesions alone. These data are supported by the presence of diffuse vertebral infiltration on MRI in 2 patients in the early progressors group and none in the late progressors group. Furthermore, the only significant factor associated with early progression was a higher serum calcium level, but, within the normal range. This suggests that these patients with SM and osteoporotic-like VF may have plasma cell infiltrates or bone fragility favored by factors secreted by plasma cells, explaining the VF.

Our study has several limitations, the first being its retrospective nature and so data collection might be subject to potential biases. Indeed, some patient might not have benefited of a close follow-up and the time between SM and MM diagnosis could be overestimated. Moreover, bone mineral density data were often missing. However, other authors have shown the limited value of this parameter by comparing MGUS and MM patients (Golombick and Diamond, 2008). Dosages of factors such as RANKL and DKK1, known to be associated with MM bone lesions (Kyle et al., 2014), were not performed and could have helped to better interpret the significance of these osteoporotic-like lesions. Also, our search was based on ICD-10 codes of the hospital discharge report, and we could not exclude that we missed some cases.

In conclusion, patients with SM and osteoporotic-like VF often progress to symptomatic MM with CRAB criteria within 18 months in half of them. Therefore, these patients need to be followed closely in order not to miss the best time to start treatment. Further prospective studies with measurement of bone mineral density, factors associated with MM bone lesions, and close follow-up are needed to assess whether osteoporotic-like VF in the absence of previous or other apparent cause of osteoporosis should be considered a CRAB criterion and warrant initiation of treatment.

The following is the supplementary data related to this article.

Table S1 Cause of secondary osteoporosis (other than multiple myeloma). Table S1

## CRediT authorship contribution statement

**Kevin Chevalier:** Writing – original draft, Resources, Methodology, Investigation, Formal analysis. **Sabrina Hamroun:** Writing – original draft, Formal analysis. **Samuel Bitoun:** Writing – review & editing, Writing – original draft, Methodology. **Julien Henry:** Writing – review & editing. **Christian Roux:** Writing – review & editing, Methodology. **Karine Briot:** Writing – review & editing, Methodology. **Rakiba Belkhir:** Writing – review & editing, Methodology. **Xavier Mariette:** Writing – review & editing, Validation, Supervision. **Raphaèle Seror:** Writing – review & editing, Validation, Supervision.

## Declaration of competing interest

KC has no conflict of interest.

SH has no conflict of interest.

SB has received grant from SERVIER, unrelated to his work.

JH has no conflict of interest.

KB has Honoria and research grant from Amgen, Theramex, and Kyowa Kirin, unrelated to his work.

RB has received Honoria from Astra Zaneca, unrelated to his work.

RC has received Honoria and research grant from ALEXION and research grant from REGENRON, unrelated to his work.

XM has received Honoria from BMS, Galapagos, GSK, Novartis, Pfizer, unrelated to his work.

RS received consulting fees or honoraria from GSK, Bristol Myer Squib, Boehringer, Janssen, Amgen, Pfizer and Roche, travel fees from Amgen and GSK, unrelated to his work.

## Data Availability

Data will be made available on request.
