# Supplementary material for: High rate of progression to symptomatic multiple myeloma in patients with smoldering myeloma and isolated osteoporotic vertebral fracture
Source: Bone Rep. 2024 Mar 25;21:101755. doi: 10.1016/j.bonr.2024.101755 (PMC10987890; doi:10.1016/j.bonr.2024.101755)
Supplement: Table S1 — Cause of secondary osteoporosis (other than multiple myeloma). [file mmc1.docx]

**Supplementary material**

**Table s1 – Cause of secondary osteoporosis (other than multiple myeloma)**

| Cause | Number of patients |
| --- | --- |
| Hyperthyroidism | 1 |
| Glucocorticoids | 1 |
| Premature menopause | 1 |
| Testicular insufficiency | 2 |
